# System X_c_^−^/GSH/GPX4 *axis*: An important antioxidant system for the ferroptosis in drug-resistant solid tumor therapy

**DOI:** 10.3389/fphar.2022.910292

**Published:** 2022-08-29

**Authors:** Feng-Jiao Li, Hui-Zhi Long, Zi-Wei Zhou, Hong-Yu Luo, Shuo-Guo Xu, Li-Chen Gao

**Affiliations:** ^1^ School of Pharmacy, University of South China, Phase I Clinical Trial Centre, The Affiliated Changsha Central Hospital, Hengyang Medical School, University of South China, Changsha, China; ^2^ Hunan Provincial Key Laboratory of Tumor Microenvironment Responsive Drug Research, Hengyang, China

**Keywords:** system Xc -/GSH/GPX4 axis, ferroptosis, drug resistance, solid tumor, therapy

## Abstract

The activation of ferroptosis is a new effective way to treat drug-resistant solid tumors. Ferroptosis is an iron-mediated form of cell death caused by the accumulation of lipid peroxides. The intracellular imbalance between oxidant and antioxidant due to the abnormal expression of multiple redox active enzymes will promote the produce of reactive oxygen species (ROS). So far, a few pathways and regulators have been discovered to regulate ferroptosis. In particular, the cystine/glutamate antiporter (System X_c_^−^), glutathione peroxidase 4 (GPX4) and glutathione (GSH) (System X_c_^−^/GSH/GPX4 axis) plays a key role in preventing lipid peroxidation-mediated ferroptosis, because of which could be inhibited by blocking System X_c_^−^/GSH/GPX4 axis. This review aims to present the current understanding of the mechanism of ferroptosis based on the System X_c_^−^/GSH/GPX4 axis in the treatment of drug-resistant solid tumors.

## Introduction

Ferroptosis, as a regulatory cell death (RCD), has been a research hotspot in the past decade (Galluzzi et al., 2018). The concept of ferroptosis was first proposed by Dr. Brent R Stockwell’s group in 2012, who discovered this new cell death pattern that differs from apoptosis, necrosis and autophagy (Dixon et al., 2012). Ferroptosis is thought to be driven by the imbalance between oxidative stress and antioxidant systems (Kuang et al., 2020). Furthermore, ferroptosis can be activated through extracellular (e.g., by inhibiting System X_c_^−^), and intracellular (e.g., by inhibiting GPX4) pathways (Tang D. et al., 2021). The lipid peroxidation is a free radical-driven reaction that primarily affects polyunsaturated fatty acids (PUFAs) in cell membranes, the product of which gradually increases during the ferroptotic cell death, from the initial lipid hydroperoxides (LOOHs) to the later production of malondialdehyde (MDA) and 4-hydroxynenoal (Kagan et al., 2017). Therefore, ferroptosis leads to the cell membrane rupture and finally death mainly by iron overload and disorders of the antioxidant system. Ferrous ion has the effect of mediating ROS production and enzyme activity during lipid peroxidation, which can produce a large number of ROS through the Fenton reaction (Dixon et al., 2012), and it also increase the activity of lipoxygenase (LOX) or EGLN prolyl hydroxylases, which are responsible for lipid peroxidation and oxygen homeostasis (Chen P. et al., 2020), so excessive accumulation of iron can result in oxidative damage to cells.

The System X_c_^−^/GSH/GPX4 axis is an important antioxidant system ferroptosis. Most ferroptosis inducers such as Erastin and RSL3, are inhibitors of System X_c_^−^/GSH/GPX4 axis, which provide a good basis for us to understand the role of different antioxidants in inhibiting ferroptosis (Kuang et al., 2020).

Since tumor cells with certain oncogenic mutations are very sensitive to ferroptosis, triggering ferroptosis may also have significant therapeutic potential for ferroptosis-sensitive tumor cells (Chen P.-H. et al., 2020). Considering the role of ferroptosis in RCD, ferroptosis might play an important role in tumorigenesis and tumor development. Moreover, drug-resistant tumor cells are more sensitive to lipid peroxidation, and inhibitors of the System X_c_^−^/GSH/GPX4 axis have been shown to be fatal in host cells. Zhang et al. found that inhibiting the GPX4 with RAS-selective lethal small molecule 3 (RSL3) enhances the antitumor effect of cisplatin (Zhang et al., 2020). Other studies have shown that GPX4 inactivation may increase susceptibility to ferroptosis in renal clear cell carcinoma by increasing lipid peroxidation (Zou et al., 2019). More recently, Ubelacker et al. found that GPX4 inhibitors make melanoma difficult to spread through blood vessels (Ubellacker et al., 2020). Therefore, induction of ferroptosis has emerged as a therapeutic strategy to trigger cancer cell death for drug-resistant solid tumors. Considering these advantages, ferroptosis is expected to become a promising therapeutic strategy for drug-resistant tumors in the near future, either alone or in combination. In this review, we summarize the regulation of the System X_c_^−^/GSH/GPX4 axis, a major antioxidant system in ferroptosis, and its potential role in drug-resistant solid tumors therapy, and also conclude with a summary of drugs, compounds and nanoparticles targeting this axis that have been studied in recent years.

## The antioxidation of system X_c_^−^/GSH/GPX4 *axis* in ferroptosis

### System X_c_^−^: The pivotal upstream node of system Xc^−^/GSH/GPX4 *axis*

**FIGURE 1:**
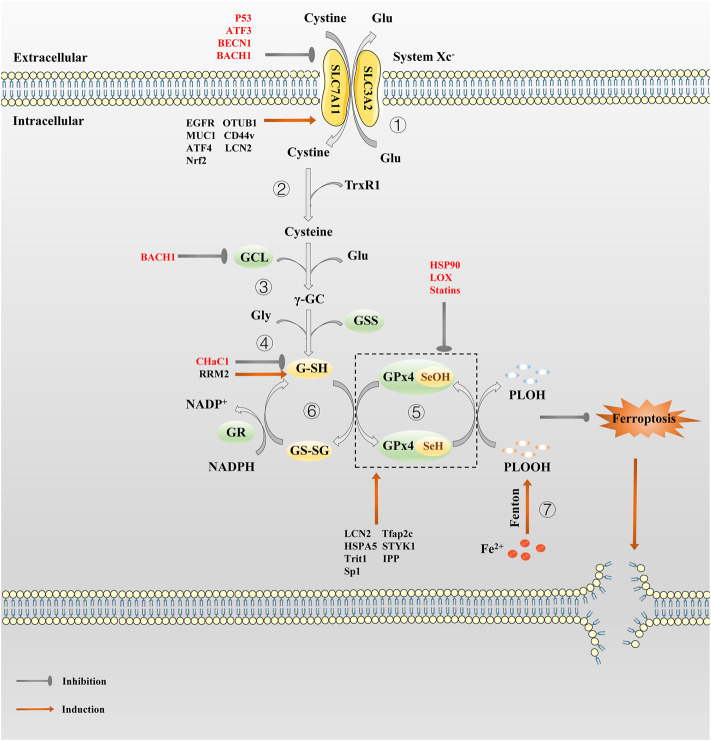
The regulation pathway of System Xc^−^/GSH/GPx4 in ferroptosis. ① System Xc^−^ transport cystine into the cell and reverse Glu out of the cell in a 1:1 ratio. ② Cystine absorbed by System Xc^−^ is reduced to cysteine by G-SH or TrxR1. ③ Then GCL links cysteine and glutamate to produce γ-GC. ④ γ-GC and Gly are catalyzed by GSS to produce G-SH. ⑤In the catalytic cycle of GPx4, the GPx4-SeH is oxidized by the P-LOOH to GPx4-SeOH, while G-SH can reduce -SeOH and further activates GPx4, releasing GS-SG to prevent GPx4 from being inactivated. ⑥GS-SG is reduced to G-SH under the action of GR and Coenzyme NADPH. Since P-LOOH is reduced to PLOH by GPx4-SeH, ferroptosis is inhibited. ⑦Fe^2+^ can produce a large number of PLOOH through the Fenton reaction.

By far, System X_c_^−^ is studied widely (Lewerenz et al., 2013). It is a chloride-dependent and sodium-independent antiporter of Cys and Glu, consisting of catalytic subunit xCT/Solute Carrier Family 7 Member 11 (SLC7A11) and regulatory subunit 4F2 (4F2hc)/Solute Carrier Family 3 Member 2 (SLC3A2) connected by disulfide bonds (Gochenauer and Robinson, 2001; Patel et al., 2004). Activation of SLC7A11 expression enables cells to restore redox homeostasis and maintain survival under stressful conditions such as oxidative stress, amino acid starvation, metabolic stress, and genotoxic stress (Koppula et al., 2018). System X_c_^−^ is driven by concentrations gradient from extracellular Cys and intracellular Glu, transporting Cys and Glu in a 1:1 ratio (Figure 1). Cys absorbed by System X_c_^−^ is reduced to cysteine by G-SH or thioredoxin reductase 1 (TrxR1), which then is used for GSH biosynthesis (Mandal et al., 2010; Conrad and Sato, 2012). Since cysteine is a speed-limiting substrate for GSH biosynthesis, and GSH is the main antioxidant in mammalian cells, hindering intracellular cysteine and GSH levels can directly affect the activity of GPX4, which can easily induce ferroptosis. There are many compounds that interfere with System X_c_^−^, such as Erastin and its analogues, that can lead to cysteine deprivation, glutathione depletion, endoplasmic reticulum stress, and cell death (Dixon et al., 2014; Sato et al., 2018). System X_c_^−^ is therefore a pivotal regulatory channel of System X_c_^−^/GSH/GPX4 axis. Although intracellular Cys is not only provided by System X_c_^−^, but also by transsulfuration pathway (Hayano et al., 2016) and the neutral amino acid transporter (Conrad and Sato, 2012). If System X_c_^−^ is dysfunctional, it will still lead to intracellular Cys deficiency, and GSH depletion, making the cell highly sensitive to ferroptosis. As an important target for inducing ferroptosis, System X_c_^−^ can provide a new direction for the treatment of drug-resistant solid tumors.

### GSH: The main cofactor of system X_c_^−^/GSH/GPX4 *axis*

GSH is a core antioxidant that is formed by condensation of Glu, Cys, and Gly. GSH, as the main cofactor of GPX4, plays the role of an electron donor or receptor by conversion between G-SH and GS-SG, making it important to fight oxidative stress (Deponte, 2013). The direct effect of GSH on ferroptosis is demonstrated by the use of Erastin, which activates a morphologically identical form of cell death caused by the lack of GPX4 in sensitive cells by lowering GSH levels (Dixon et al., 2012). Inhibiting the synthesis and utilization of GSH is a classic method to induce ferroptosis (Yang et al., 2014). What’s more, the role of GSH in ferroptosis depends mainly on GPX4. GPX4 can catalyze the reduction of phospholipid hydroperoxide (PLOOH) to the corresponding hydroxyl derivatives (Imai and Nakagawa, 2003). In the catalytic cycle of GPX4, the -SeH (the main active group of GPX4) is oxidized by the PLOOH to selenic acid (-SeOH), while G-SH can reduce -SeOH and further activates GPX4, releasing GS-SG to prevent GPX4 from being inactivated (Ingold et al., 2018). The GSH depletion induce many cancer cells to ferroptosis, such as pancreatic tumor (Badgley et al., 2020), liver cancer (Chen et al., 2019; Yang Y. et al., 2020), prostate cancer (Qin et al., 2021), breast cancer (Yang J. et al., 2021) etc. It is also possible to produce an effect similar to GPX4 inactivation by inhibiting the GSH synthesis or antagonizing GSH.

### GPX4: The core downstream antioxidant of system X_c_^−^/GSH/GPX4 *axis*

As the cornerstone of antioxidant defense, GPX4, a phospholipid peroxidation inhibitor, is the only glutathione peroxidase used for intracellular lipid peroxide reduction (Ursini et al., 1982). In the presence of GSH, GPX4 continuously reduce PLOOHs, and if GSH depletion or GPX4 inactivation, intracellular ferrous ions induce ferroptosis by breaking down PLOOHs to cause lipid peroxidation (Ursini and Maiorino, 2020). In early studies, selective knockout of the GPX4 gene in hippocampal neurons promotes neurodegeneration and cell death in a non-apoptotic manner, which provide preliminary evidence for GPX4 as a particular regulator of ferroptosis (Seiler et al., 2008). Subsequently, Stockwell’s team demonstrated that GPX4 is a key upstream regulator of ferroptosis in 2014 (Friedmann Angeli et al., 2014; Yang et al., 2014). Well-balanced GSH levels and GPX4 function are significant for maintaining intracellular redox homeostasis. Whether knocking out the GPX4 gene or inhibiting its activity, it can eventually lead to a redox homeostasis imbalance that can have a fatal effect on normal or tumor cells. Photosensitive cells (Ueta et al., 2012), renal tubular cells (Friedmann Angeli et al., 2014), CD8^+^ T cells (Matsushita et al., 2015), vascular endothelial cells (Wortmann et al., 2013), hepatocytes (Carlson et al., 2016), sperm cells (Imai et al., 2009), etc. will be highly sensitive to ferroptosis in result of GPX4 inactivation. In addition, inhibiting GPX4 also promote ferroptosis in some malignant tumor cells such as pancreatic ductal adenocarcinoma (Dai et al., 2020) and colorectal cancer cells (Sui et al., 2018), etc. However, as early as the 1996 study by Imai et al. revealed that GPX4 overexpression prevents basophilic leukemia cells in mice from death caused by oxidative damage (Imai et al., 1996), thus, theoretically, GPX4 overexpression could make cells resistant to oxidative stress-induced ferroptosis. Hence, taking GPX4 as a target is of great help for the treatment of drug-resistant tumors.

## The regulation of system X_c_^−^/GSH/GPX4 *axis* in ferroptosis

As the main antioxidant system against ferroptosis, the role of System X_c_^−^/GSH/GPX4 axis is crucial in the treatment of various types of drug-resistant solid tumors, so an in-depth understanding of the regulatory mechanism of each node on this axis is of great significance for developing effective strategies for System X_c_^−^/GSH/GPX4 axis targeted treatment of drug-resistant solid tumors (Figure 1).

### The regulation of system X_c_^−^ in ferroptosis

As mentioned before, the activity of System X_c_^−^ is mainly determined by SCL7A11 (Sato et al., 1999). The low expression of SCL7A11 reduces System X_c_^−^ activity, resulting in oxidative stress-mediated ferroptosis; conversely, the overexpression of SCL7A11 enhance cell resistance to ferroptosis, which is also one of the reasons for drug resistance in tumor cells (Huang et al., 2005; Dai et al., 2007; Lin et al., 2020). Therefore, the regulation of SCL7A11 is of great importance in ferroptosis resistance. To ensure the proper function of SLC7A11 in maintaining redox homeostasis, the expression and activity of SLC7A11 is strictly regulated by a variety of mechanisms, including transcriptional and epigenetic regulator-mediated transcriptional regulation, as well as post-transcriptional regulatory mechanisms.

Activating transcription factor 4 (ATF4) and nuclear factor erythroid 2-related factor 2 (Nrf2) are the two main transcription factors that mediate stress-induced SLC7A11 transcription. Under various stress conditions, such as amino acid starvation, endoplasmic reticulum stress, hypoxia and virus infection, ATF4 (a member of the ATF/CREB transcription factor family) is mainly induced by mRNA translation (Pakos-Zebrucka et al., 2016). Under the stress of amino acid deprivation, the expression of SLC7A11 is largely mediated by ATF4 (Sato et al., 2004). The translation of ATF4 mRNA increases through the general control non-derepressible-2 (GCN2)-eukaryotic initiation factor 2α (eIF2α) signal axis. Subsequently, ATF4 binds to the amino acid response element (AARE) of gene promoter to further promote the transcription of genes involved in amino acid metabolism and stress response, such as SLC7A11, so that cells can cope with amino acid constraints (Kilberg et al., 2009). SLC7A11 protects cells from ferroptosis caused by cystine starvation (Stockwell and Jiang, 2020). A study has shown that ATF4 can stimulate the transcription of SCLS7A11 and promote tumor angiogenesis (Chen et al., 2017a).Another transcription factor, Nrf2, which promotes SLC7A11 transcription, is a key regulator of antioxidant response. Under non-stress conditions, Nrf2 maintained at a low level via the proteasome degradation mediated by Kelch-like ECH-associated protein 1 (Keap1); instead, Nrf2 dimerizes with members of the small Maf family and binds to the antioxidant response elements (AREs) located in the regulatory region of the cell defense enzyme gene, relieving the inhibition of Keap1 and activating the transcription of cellular protective genes such as SLC7A11 to play an antioxidant role (Ma, 2013). Subsequent studies have proved that activation of Nrf2 and inhibition of Keap1 lead to SLC7A11 upregulation, thereby promoting resistance to ferroptosis (Chen et al., 2017b; Fan et al., 2017).The expression of SLC7A11 can also be inhibited by transcription factors such as Tumor protein p53 (p53) and Activating transcription factor 3 (ATF3). SLC7A11 is a transcription inhibition target of p53 (Jiang et al., 2015; Jennis et al., 2016). Under different ferroptosis induction conditions, p53 promotes ferroptosis partly by inhibiting SLC7A11 expression, while p53 deficiency promotes ferroptosis resistance by SLC7A11 upregulation. ATF3 inhibits SLC7A11 by binding to the SLC7A11 promoter in a p53-independent manner, and promotes Erastin-induced ferroptosis by SLC7A11 downregulation (Wang L. et al., 2020).

Epigenetic regulation of transcription is crucial to control intracellular homeostasis and development. Recent studies have revealed the key role of epigenetic regulation of SLC7A11 transcription in the control of ferroptosis. It was recently pointed out that the anti-oncogene BRCA1-Associated Protein 1 (BAP1) deubiquitinates the H2Aub portion of the SLC7A11 gene promoter and represses SLC7A11 expression, thereby limiting Cys uptake and increasing ferroptosis sensitivity; on the contrary, the lack of BAP1 in cancer cells leads to SLC7A11 upregulation and ferroptosis resistance (Zhang et al., 2018). In addition, p53 promotes nuclear translocation of Ubiquitin Specific peptidase 7 (USP7), and USP7 removes ubiquitin from H2Bub, resulting in a decrease in H2Bub occupation on the SLC7A11 promoter, resulting in SLC7A11 transcriptional inhibition (Wang Y. et al., 2019). Moreover, a histone H3 lysine 9 (H3K9) demethylase KDM3B has been reported to be involved in transcriptional regulation of SLC7A11 (Wang Y. et al., 2020). Overexpression of KDM3B decreased H3K9 methylation (related to transcriptional inhibition) and upregulated the expression of SLC7A11, thus enhancing resistance to Erastin-induced ferroptosis. Bromodomain-containing protein 4 (BRD4) function as genetic readers of histone acetyl lysine residues to regulate gene transcription. BRD4 inhibitor JQ1 can induce ferroptosis both *in vitro* and in Xenograft model; JQ1 therapy or BRD4 gene knockout can down-regulate ferroptosis regulators including SLC7A11 (Sui et al., 2019). Another key epigenetic mechanism that controls gene transcription involves chromatin remodeling mediated by the SWI/SNF complex, which has been revealed to be related to SLC7A11 transcriptional regulation (Ogiwara et al., 2019). Mechanically, ARID1A is a component of the SWI/SNF complex, which binds to the SLC7A11 promoter and promotes NRF2-mediated SLC7A11 transcriptional activation.

SLC7A11 can be regulated by different post-translation mechanisms. Previous studies have shown that Epidermal Growth Factor Receptor (EGFR), CD44v (an adhesion molecule expressed in cancer stem-like cells) and OTU deubiquitinase, ubiquitin aldehyde-binding 1 (OTUB1), control and stabilize SCL7A11 expression, facilitating Cys uptake by tumor cells (Ishimoto et al., 2011; Tsuchihashi et al., 2016; Liu et al., 2019). Among them, CD44v hastens the interaction of OTUB1-SCL7A11, while inhibition of OTUB1 accelerates the degradation of SLC7A11 (Liu et al., 2019). In addition, transmembrane mucin glycoprotein Mucin 1 (MUC1) binds directly to CD44v, enhancing the stability of SCL7A11 and thus controlling GSH levels (Hasegawa et al., 2016). SLC7A11 can also be inactivated by AMPK-mediated phosphorylation of BECN1, which leads to the formation of BECN1-SLC7A11 complex (Kang et al., 2018; Song et al., 2018). Mechanistic investigation identified SLC7A11 was a direct target of METTL14. Both *in vitro* and *in vivo* assay demonstrated that METTL14 induced N6 -methyladenosine (m^6^A) modification at 5′UTR of SLC7A11 mRNA, which promotes SLC7A11 mRNA stability and upregulates its expression by inhibiting the deadenylation process, enhancing ferroptosis resistance (Fan et al., 2021; Liu L. et al., 2022; Hill and Liu, 2022; Peng and Mo, 2022). Additionally, some small molecular inhibitors such as Erastin (Dixon et al., 2014). Piperazine Erastin (PE) and imidazole ketone Erastin (IKE) (Yang et al., 2014; Larraufie et al., 2015), also have good capacity to inactive SLC7A11. In general, interfering with SCL7A11 expression modulates System X_c_^−^ activity during ferroptosis, thereby regulating cancer cell replication, tissue invasion, and metastasis.

### The regulation of GSH in ferroptosis

GSH is a key antioxidant against ferroptosis. The synthesis and degradation of GSH play an important part in GSH abundance. Under physiological conditions, GSH synthesis relies on two key enzyme (glutamate cysteine ligase (GCL) and glutathione synthetase (GSS)) and the availability of cysteine (Lu, 2009, 2013). GCL consists of Glutamate-Cysteine ligase Catalytic Subunit (GCLC) and Modifier Subunit (GCLM). Genetic inhibition of GCLC enhances ferroptosis due to metabolic stress, including cystine starvation (Gao et al., 2015).GCL links cysteine and Glu to produce γ-glutamylcysteine (γ-GC) which is catalyzed to G-SH by GSS. Thus, those that inhibit System X_c_^−^ activity are able to inhibit GSH biosynthesis through Cys depletion. Depletion of amino acids other than Cys also lead to GSH depletion (Tan et al., 1998; Sato et al., 2004). Mammals usually rely solely on extracellular uptake as the primary source of Cys, but some mammals also use cystathionine γ-lyase (CGL)-mediated cystathionine-cleavage in the transsulfuration pathway as a surrogate Cys source if System X_c_^−^ is blocked (Shimada and Stockwell, 2016). As an important transcription factor, Nrf2 can not only regulate SLC7A11 transcription, but also up-regulate the expression of GSS and GCLC, which are key rate-limiting enzymes for GSH synthesis (Dodson et al., 2019). Additionally, Nrf2 promotes GSH efflux, which is an unexpected regulator of ferroptosis sensitivity, while Nrf2 inhibition can act synergistically with ferroptosis inducers (Sun et al., 2016a; Sun et al., 2016b; Cao et al., 2019). In addition to inhibiting cellular Cys uptake, ferroptosis can also be achieved by consuming Cys in extracellular. One study demonstrated that an engineered and pharmacologically optimized human cyst(e)inase enzyme that consistently depletes extracellular Cys pools (Cramer et al., 2017). Genome-wide siRNA screening reveals that knockdown of cysteinyl-tRNA synthetase gene activates transulfuration pathway and inhibits ferroptosis induced by System X_c_^−^ inhibitors (e.g. Erastin) (Hayano et al., 2016).

Irreversible inhibitors of GCL, buthionine sulfoxide (BSO) and Cysteine sulfonimide, reduce GSH formation and further inhibit GPX4 activity, promoting ferroptosis alone or enhancing sensitivity to ferroptosis (Yang et al., 2014). Ribonucleotide reductase regulatory subunit M2 (RRM2, a structural unit essential for DNA replication and repair) maintains intracellular GSH by protecting GLC from degradation to exert an anti-ferroptosis effect (Yang Y. et al., 2020). Moreover, ChaC Glutathione Specific Gamma-Glutamylcyclotransferase 1 (CHaC1) also regulates GSH degradation and has been detected to be downregulated in some tumor cells (Hong et al., 2021; Xiao et al., 2022). CHaC1 upregulation promotes GSH degradation and leads to GSH depletion (Chen M.-S. et al., 2017). A recent study indicated that BTB and CNC homology 1 (BACH1, a heme-binding transcription factor required for the regulation of oxidative stress and metabolic pathways associated with heme and iron), suppresses GSH synthesis pathway-related genes such as GCLM and GCLC to reduce GSH and regulate ferroptosis (Igarashi and Watanabe-Matsui, 2014; Nishizawa et al., 2020).

### The regulation of GPX4 in ferroptosis

GPX4 is an important arsenic protein in mammals that directly reduces PLOOHs during ferroptosis, and its redox activity depends on the 21st amino acid selenocysteine (Sec) (Ingold et al., 2018). As a key downstream antioxidant enzyme of System X_c_^−^/GSH/GPX4 axis, GPX4 is often used to develop ferroptosis inducers as an effective target. The activity of GPX4 is regulated by the selenium potency, which is thought to be a constraint on susceptibility to ferroptosis (Hatfield et al., 1991; Cardoso et al., 2017). The mevalonate pathway provides selenium for GPX4 maturation, but statins can block this pathway (Chen and Galluzzi, 2018). Except for selenium, isopentenyl pyrophosphate (IPP) produced by the mevalonate pathway also facilitates GPX4 synthesis (Shimada et al., 2016; Ingold et al., 2018). Additionally, GSH is a main cofactor of GPX4, so GSH regulation can also indirectly modulate GPX4 (Maiorino et al., 2018).

Regulation of GPX4 expression includes multiple mechanisms, such as gene transcription and post-translational modification, which significantly affect the level of lipid peroxidation in tissue damage. The upstream regulation of GPX4 mainly include transcription factors such as Nrf2 (Dodson et al., 2019), MYB (Hao et al., 2017), Tfap2c (Alim et al., 2019), Sp1 (Alim et al., 2019), Lipocalin 2 (LCN2) (Chaudhary et al., 2021), mTORC1 (Saxton and Sabatini, 2017; Zhang Y. et al., 2021), and NUAK2 (Singh et al., 2022). GPX4 is target gene of Nrf2. Nrf2 inhibition reverses resistance to GPX4 inhibitor-induced ferroptosis in head and neck cancer (Shin et al., 2018). Selenium-induced transcription factors Tfap2c and Sp1 upregulate GPX4 to prevent ferroptosis-associated cerebral hemorrhage. While mTORC1 is a key signaling node that integrates multiple environmental signals to modulate protein (e.g., GPX4) synthesis, and its inactivation reduces GPX4 and sensitizes tumor cells to ferroptosis. In contrast, LCN2, a key protein regulating iron homeostasis, inhibit ferroptosis by stimulating GPX4 and SCL7A11 expression. Furthermore, knockdown of tRNA isopentenyltransferase 1 (Trit1, an essential selenoprotein synthesis enzyme) reduces selenoproteins expression (Fradejas et al., 2013). Post-translational modification affects the stability of GPX4 proteins by modulating the degradation of GPX4. The post-translational modifications of GPX4 mainly include succination, ubiquitination and alkylation. Succination is a non-enzymatic, irreversible protein modification mediated by fumaric acid (an intermediate product of Krebs cycle in mitochondria) (Alderson et al., 2006). Fumaric acid binds to the thiol group of cysteine residues in the absence of enzymes to form thioether bonds. It has been found that intracellular fumaric acid aggregation causes succination of GPX4 at cysteine 93, resulting in a decrease in GPX4 activity and sensitizing cancer cells to ferroptosis inducers (Kerins et al., 2018). Ubiquitination is the process by which ubiquitin is added to the lysine residue of the substrate protein by enzymes E1, E2, and E3, which eventually leads to the degradation of the protein by the proteasome. And deubiquitinase (DUB) stabilizes intracellular protein structure by removing ubiquitin chains from ubiquitinated proteins. A recent study by Liu’s team demonstrated that the broad-spectrum DUB inhibitor palladium pyrithione complex (PdPT) promote GPX4 degradation, but proteasome inhibitor bortezomib reverses its effect (Yang L. et al., 2020). Another study showed that a bartoldine derivative, DMMOCPTL, binds directly to selenosecysteine 46 of GPX4, resulting in ubiquitination of GPX4 in triple-negative breast cancer cells (Ding et al., 2021). Alkylation is the chemical process of introducing one or more alkyl groups into a protein or compound. Small molecule inhibitors such as RSL3 and ML162 mediate alkylation on GPX4 by binding to the selenium cysteine 46 residue by electrophilic alkyl chloride moieties (Eaton et al., 2020; Vučković et al., 2020). Heat shock protein A family member 5 (HSPA5, a molecular chaperone in the endoplasmic reticulum) binds directly to GPX4 to prevent its degradation (Zhu S. et al., 2017). They also found that LOX catalyzes the covalent inhibition of selenocysteine in GPX4 (Yang et al., 2016).

What’s more, There are also some other small molecule regulators that interfere GPX4 such as FIN56 (Shimada et al., 2016), FINO2 (Gaschler et al., 2018), ML210 (Eaton et al., 2020), JKE-1674 (Eaton et al., 2020), JKE-1716 (Eaton et al., 2020), NSC144988 (Stockwell and Jiang, 2020) and PKUMDL-LC-101 series (see in (Li et al., 2019)).

## Potential roles of targeting system X_c_^−^/GSH/GPX4 *axis* in drug-resistant solid tumor

**FIGURE 2:**
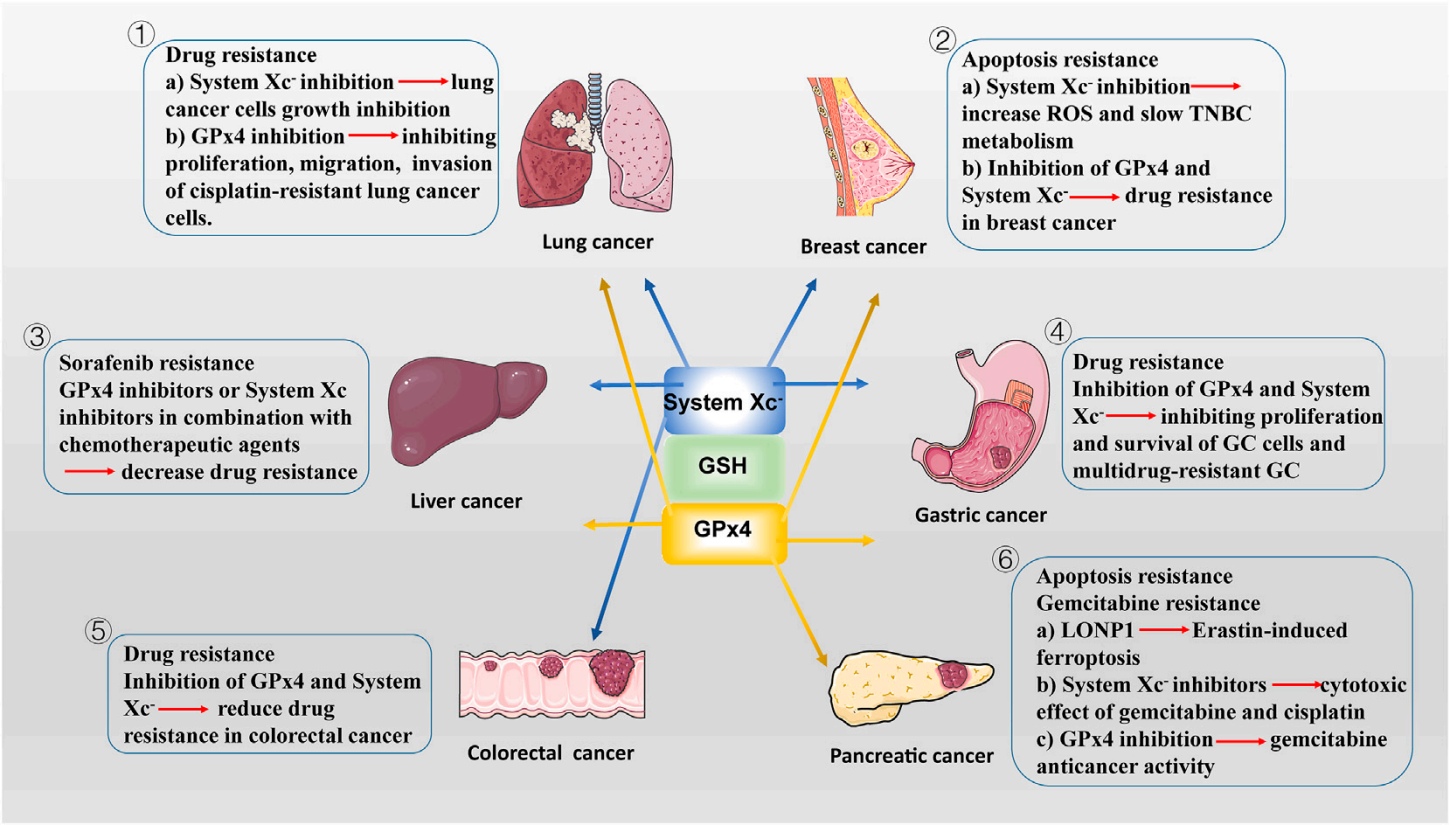
The potencial roles of System Xc-/GSH/GPx4 in drug-resistant solid tumor. ① SLC7A11, highly expressed in NSCLC, is a potential target for ferroptosis. SLC7A11 downregulation lead to ferroptosis of lung cancer cells and inhibit their growth. In addition to SLC7A11, lung cancer cell also exhibits high GPx4 expression. GPX4 inhibitor limits proliferation, migration, and invasion of cisplatin-resistant lung cancer cells. ② Drug-resistant breast cancer cells are dependent on GPX4 and SLC7A11. SLC7A11 is upregulated in one-third of TNBC cells *in vivo*, and inhibiting System Xc-activity increases intracellular ROS levels and slows TNBC metabolism. Inhibition of GPX4 and/or System Xc-may be a potential measure to overcome drug resistance in breast cancer. ③ The main regulatory mediators mediating the ferroptotic response in HCC cells have been identified as System Xc-and GPX4. blocking System Xc -/GSH/GPX4 axis in combination with chemotherapeutic agents (e.g., sorafenib) provides new ideas for treatment of drug-resistant HCC. ④ GPx4 is lowly expressed in GC cells, making them more susceptible to ferroptosis than normal intestinal cells. Reducing the expression of GPX4 and System Xc-inhibiting the proliferation of GC cells and multidrug-resistant GC. ⑤ In CSCs, SLC7A11 is extremely expressed, with high GSH levels and low ROS levels, leading to their extreme vulnerability to ferroptosis. Similar to GC cells, targeting the System Xc-/GSH/GPX4 axis is an effective way to inhibit the growth of drug-resistant colorectal cancer. ⑥ LONP1 inhibits Nrf2-mediated GPX4 gene expression, thereby promoting Erastin-induced ferroptosis in human PDAC cells. The use of System Xc-inhibitors enhanced the cytotoxic effect of gemcitabine and cisplatin on PDAC cell lines. Gemcitabine resistance was associated with GPx4 upregulation in PDAC cells. Inhibition of GPX4 activity or induction of GPX4 degradation can restore or enhance the anticancer activity of gemcitabine *in vitro* or in xenogeneic PDAC models.

Chemotherapy are by far one of the most commonly used methods to treat malignant tumors, but the continuous overdose of chemotherapeutic drugs has led to varying degrees of resistance and increased aggressiveness of some tumors. Ferroptosis is a RCD caused by intracellular iron accumulation combined with disruption of antioxidant systems, such as GPX4 inactivation and GSH depletion, and subsequent accumulation of toxic lipid peroxides (Dixon et al., 2012; Yang et al., 2014). Importantly, drug-resistant tumor are more sensitive to lipid peroxidation, which undoubtedly makes, the combination of System X_c_^−^/GSH/GPX4 axis-based ferroptosis inducers with chemotherapeutic agents may become a new strategy for the treatment of drug-resistant solid tumors (Viswanathan et al., 2017). There is also growing evidence that disruption of antioxidant systems in ferroptosis contributes to the anticancer treatment of several forms of drug-resistant solid tumors (Figure 2).

### Lung cancer

SLC7A11, highly expressed in NSCLC, is a potential target for ferroptosis (Baek et al., 2012). SLC7A11 overexpression promote lung cancer cell metastasis and proliferation *in vivo* and *in vitro* by mediating cellular uptake of cysteine and reducing ROS production; conversely, SLC7A11 downregulation lead to ferroptosis (Liu et al., 2020; Chen M. et al., 2021).Meanwhile, lung cancer cell also developed ferroptosis resistance by some mechanisms to enhance the expression of SLC7A11. It has been reported that the RNA-binding protein RBMS1 bridges the 3′ and 5′-UTR of SLC7A11 to enhance its expression by interacting directly with the translation initiation factor eIF3d. And RBMS1 ablation inhibits the translation of SLC7A11, reduces SLC7A11-mediated cystine uptake, and promotes ferroptosis (Zhang W. et al., 2021). In addition, a new miRNA, miR-27a-3p, regulates ferroptosis by directly targeting SLC7A11 in NSCLC cells. miR-27a-3p overexpression leads to SLC7A11 inhibition by direct binding to its 3′-UTR, followed by a reduction of erastin-induced ferroptosis. In contrast, miR-27a-3p inhibiton increases sensitivity of NSCLC cells to erastin (Lu et al., 2021). Moreover, SLC7A11 is also upregulated in lung cancer stem cell-like cells and activated by the cell transcription factor SOX2 (Wang X. et al., 2021). Tumors with higher SOX2 expression are more resistant to ferroptosis, and the expression of SLC7A11 is positively correlated with SOX2 in mouse and human lung cancer tissues (Wang X. et al., 2021).

In addition, Kirsten Rat Sarcoma (KRAS)-mutant lung cancer tumor progression is closely associated with SLC7A11 expression. In several preclinical lung cancer mouse models, treatment of KRAS-mutant lung adenocarcinoma (LUAD) with HG106 (a potent System X_c_^−^ inhibitor), significantly inhibited tumor growth and prolonged survival (Hu et al., 2020). The latest researches show that m^6^A is associated with regulating sensitivity of LUAD to ferroptosis. The m^6^A reader YT521-B homology containing 2 has been identified to inhibit LUAD tumorigenesis by suppressing SLC7A11 and SLC3A2 (Ma et al., 2021). And methyltransferase-like 3, the main catalyst of m^6^A, mediate m^6^A modification to stabilize SLC7A11 mRNA and promote its translation, which enhances LUAD cell proliferation and inhibits cell ferroptosis (Xu Y. et al., 2022). Additionally, *in vivo*, Circular RNA CircP4HB expression levels are increased in LUAD, which inhibits ferroptosis by regulating miR-1184/SLC7A11-mediated GSH synthesis and, therefore protected LUAD cells from ferroptosis induced by erastin (Pan et al., 2022).

In addition to SLC7A11, lung cancer cell also exhibits high GPX4 expression. GPX4 promotes proliferation and ferroptosis resistance in lung cancer, while GPX4 inhibitor RSL3 limits proliferation, migration, and invasion of cisplatin-resistant A549 cells (Deng et al., 2021). Ni et al. found that GPX4 was upregulated because of enhanced activation of mTORC1 in lapatinib resistant NSCLC cells (Ni J. et al., 2021). Inhibition of mTORC1 leads to the downregulation of GPX4. Further *in vivo* experiments also showed that the silencing of GPX4 enhanced the anti-cancer effect of lapatinib by promoting ferroptosis. A Circular RNA CircDTL acting as an oncogene, was found to be upregulated and exerts its effects via the miR-1287-5p/GPX4 axis in NSCLC (Shanshan et al., 2021). Knockdown of circDTL promoted both apoptosis and ferroptosis of NSCLC cells. Recently, Wang’s team discovered that the promoter region of GPX4 binds to cyclic adenosine monophosphate response element binding protein (CREB) and that this binding can be enhanced by E1A binding protein P300 (EP300), promoting tumor proliferation, migration, invasion and angiogenesis. Thus, GPX4 inactivation blocks the CREB/EP300/GPX4 axis, and these findings reveal that SLC7A11 or GPX4 inhibition sensitizes LUAD cells to ferroptosis, providing a potential therapeutic approach for this currently incurable disease (Wang Z. et al., 2021). Moreover, overexpressed serine threonine tyrosine kinase 1 (STYK1) upregulates GPX4, resulting in SW900 cells to be less sensitive to ferroptosis (Lai et al., 2019). Importantly, GPX4 was positively correlated with resistance of lung cancer cells to L-685458, lapatinib, paposilli, and topotecan, suggesting that targeting System X_c_^−^/GSH/GPX4 axis could overcome drug resistance (Zhang et al., 2020; Ni J. et al., 2021).

A number of studies have proved that some natural compounds (Table 1) such as dihydroartemisinin (Yuan et al., 2020), artesunate (Zhang Q. et al., 2021),sulforaphane (Iida et al., 2021), curcumin (Tang X. et al., 2021), bufotalin (Zhang W. et al., 2022), sanguinarine (Xu R. et al., 2022), sinapine (Shao et al., 2022), solasonine (Zeng et al., 2022), ophiopogonin B (Zhang L. et al., 2022), red ginseng polysaccharide (Zhai et al., 2022) Dihydroisotanshinone I (Wu et al., 2021).inhibits the proliferation, colony formation and induces ferroptosis of lung cancer cells by the interfering mRNA and/or protein expression and/or degradation of SLC7A11 or GPX4. Apart from natural compounds, some drugs that have been marketed (Table 1) such as Vorinostat (Zhang T. et al., 2021), Orlistat (Zhou W. et al., 2021) have also been found to act as ferroptosis inducers in lung cancer cells via inhibiting System X_c_^−^/GSH/GPX4 axis. In NSCLC, chemotherapy relies heavily on cisplatin as the first line of clinical treatment (Gridelli et al., 2018). The activation of Nrf2/SLC7A11 pathway is one of the main mechanisms of cisplatin resistance in NSCLC. Erastin or sorafenib combined with small doses of cisplatin can effectively inhibit the growth of cisplatin-resistant NSCLC cells by inhibiting the Nrf2/SLC7A11 pathway (Li et al., 2020). Conversely, SLC7A11 overexpression enhances the resistance of lung cancer cells to cisplatin (Horibe et al., 2018). Gefitinib an EGFR tyrosine kinase inhibitors resistance, was approved for second-line treatment of advanced NSCLC in 2004 and first-line treatment of patients with EGFR mutations in 2010. RSL3 combined with gefitinib inhibits the growth of gefitinib-derived persister lung cancer cells (Ishida et al., 2021). Yan et al. found that gefitinib in combination with betulin (a natural ferroptosis inducer) is a novel therapeutic approach to overcome gefitinib resistance in EGFR wild-type/KRAS-mutant NSCLC cells by inducing ferroptosis *in vitro* and *in vivo* (Yan et al., 2022). Moreover, small molecules activating ferroptosis through System X_c_^−^ inhibition or GPX4 inhibition enhance the antitumor effect of radiotherapy (Ye et al., 2020; Almahi et al., 2022) and photodynamic therapy (Han et al., 2022) in lung cancer.

**TABLE 1:** The regulatory small molecule compounds of targeting System X_c_^−^/GSH/GPX4 axis in ferroptosis.

Target	Compounds	Mechanism	Induce/Inhibit Ferroptosis	References
SLC7A11	Erastin	↓SLC7A11, ↓GSH	Induce	Dixon et al. (2014)
	PE	↓SLC7A11, ↓GSH	Induce	Yang et al. (2014)
	IKE	↓SLC7A11, ↓GSH	Induce	Larraufie et al. (2015)
	HG106	↓SLC7A11, ↓GSH, ↓ΔΨm	Induce	Hu et al. (2020)
GSH	BSO	↓GCL, ↓GSH, ↓GPX4	Induce	Yang et al. (2014)
	Cyst(e)inase	↓extracellular cystine	Induce	Cramer et al. (2017)
GPX4	PdPT	↑GPX4 degradation	Induce	Li Yang et al. (2020)
	FIN56	↑GPX4 degradation	Induce	Shimada et al. (2016)
	Rapamycin	↑GPX4 degradation	Induce	Yang Liu et al. (2021b)
	FINO2	↓GPX4	Induce	Gaschler et al. (2018)
	RSL3	↓GPX4	Induce	Vučković et al. (2020)
	ML210	↓GPX4	Induce	Eaton et al. (2020)
	ML162	↓GPX4	Induce	Shin et al. (2018)
	JKE-1674	↓GPX4	Induce	Eaton et al. (2020)
	JKE-1716	↓GPX4	Induce	Eaton et al. (2020)
	NSC144988	↓GPX4	Induce	Stockwell and Jiang, (2020)
	PKUMDL-LC-101 series	↑GPX4	Inhibit	Li et al. (2019)

Abbreviation. SLC7A11, Solute Carrier Family 7 Member 11; GSH, glutathione; GPX4, glutathione peroxidase 4; PE, piperazine erastin; IKE, imidazole ketone Erastin; ΔΨm, mitochondrial membrane potential; GCL, glutamate cysteine ligase; BSO, buthionine sulfoxide; PdPT, palladium pyrithione complex; RSL3, RAS, selective lethal small molecule 3.

### Breast cancer

Breast cancer is one of the most effective for chemotherapy in solid tumors. More than 80% of breast cancer patients require chemotherapy (Harbeck and Gnant, 2017). But most patients eventually develop drug resistance. Drug-resistant breast cancer cells are dependent on GPX4 and SLC7A11, which means they are vulnerable to ferroptosis caused by GPX4 and SLC7A11 inhibition (Hangauer et al., 2017). Selenium detoxification is critical for breast cancer survival. The micronutrient selenium is incorporated into the rare amino acid selenium cysteine through the selenium cysteine biosynthetic pathway, which is required for GPXs (Burk and Hill, 2015). The selenophosphate synthetase 2 (SEPHS2), an enzyme in the selenocysteine biosynthesis pathway, is required in cancer cells to detoxify selenide, an intermediate that is formed during selenocysteine biosynthesis (Burk and Hill, 2015). Breast and other cancer cells are selenophilic, allowing production of selenoproteins such as GPX4, protects cells against ferroptosis. However, selenide is poisonous and must be processed by SEPHS2. SEPHS2 protein levels are elevated in human breast cancer patient samples and loss of SEPHS2 impairs growth of orthortopic mammary tumor xenografts in mice (Carlisle et al., 2020). Gefitinib-targeted therapy is insufficient to inhibit triple negative breast cancer TNBC cell proliferation (McLaughlin et al., 2019). And GPX4 is increased in gefitinib-resistant cells. Silence or inhibition of GPX4 stimulate ferroptosis and enhance TNBC cell sensitivity to gefitinib (Song et al., 2020).

In 2013, Timmerman et al. identified a subset of TNBC samples as glutamine nutrient deficient by analyzing the functional metabolic profiles of 46 independently sourced breast cell lines, specifically for glutamine uptake and dependence. Tumor cells acquire Cys indirectly via peripheral glutamine using System X_c_^−^ as a carrier, SLC7A11 is upregulated in one-third of TNBC cells *in vivo*, and limiting glutamine uptake or inhibiting System X_c_^−^ activity increases intracellular ROS levels and slows TNBC metabolism (Bannai and Ishii, 1988; Timmerman et al., 2013). Further RNA sequencing analysis showed that SLC7A11 expression was upregulated in breast cancer tissues with brain metastases, suggesting a role for SLC7A11 in breast cancer metastasis (Sato et al., 2017). Yadav et al. demonstrate that miR-5096 is a tumor-suppressive miRNA that can target and downregulate SLC7A11in breast cancer cells (Yadav et al., 2021). Overexpression of miR-5096 reduces mRNA and protein levels of SLC7A11 in breast cancer cells. Virus-like particle immunotherapy or vaccines against SLC7A11 have been developed and shown to reduce the metastatic potential of breast cancer cells (Bolli et al., 2018; Donofrio et al., 2018; Ruiu et al., 2019). Both ferroptosis inducers RSL3 and sulfasalazine (SAS) inhibit GPX4 activity in breast cancer cells (Yu H, et al., 2019). These results suggest that inhibition of GPX4 and/or System X_c_^−^ may be a potential measure to overcome drug resistance in breast cancer. Nrf2 can promote drug resistance of breast cancer cells by regulating System X_c_^−^/GSH/GPX4 axis. Nrf2 upregulates the expression and activity of SLC7A11 in breast cancer cells during oxidative stress, and promotes the survival of breast cancer cells from drugs and other treatments by antagonizing ROS, while Nrf2 expression is downregulated when ROS levels are reduced (Habib et al., 2015; Mostafavi-Pour et al., 2017).

Monotherapy and combination chemotherapy are commonly used in the treatment of breast cancer. Some drugs commonly used in clinical practice have also been found to have the effect of inducing ferroptosis in breast cancer (Table 2). In addition, Metformin a commonly used hypoglycemic drugs in clinical practice, was found to promote ferroptosis in breast cancer cells by inhibiting the UFMylation of SLC7A11 and the transcription of GPX4 (Yang J. et al., 2021; Hou et al., 2021). Furthermore, one study found that Ketamine inhibit the expression of GPX4 by attenuating KAT5 on the promoter region of GPX4, repressing the enrichment of histone H3 lysine 27 acetylation and RNA polymerase II (Li H. et al., 2021). Some other small molecule compounds (Table 2) such as alloimperatorin (Zhang J. et al., 2022b), tetrandrine citrate (Yin et al., 2022), pyrrolidin-3,2′-oxindoles (Liu S.-J. et al., 2021), Saponin Formosanin C (Chen H.-C. et al., 2022) can also induce ferroptosis in breast cancer cells by interfering with the System X_c_^−^/GSH/GPX4 axis. A polymer carbohydrates *Lycium barbarum* polysaccharide effectively prevents breast cancer cell proliferation and promotes ferroptosis via the System X_c_^−^/GPX4 pathway (Du et al., 2022).

**TABLE 2:** Drugs and compounds based on System X_c_^−^/GSH/GPX4 for treatment of drug-resistant solid tumors.

Compounds	Mechanism	Cancer Cell Lines	References
Bufotalin	inhibit the expression of GPX4; facilitate the ubiquitination and degradation of GPX4	A549	Yilei Zhang et al. (2022)
Sanguinarine	Decrease the protein stability of GPX4 through E3 ligase STUB1-mediated ubiquitination and degradation of endogenous GPX4	A549 and H3122	Rongzhong Xu et al. (2022)
Sinapine	Increase intracellular Fe^2+^, lipid peroxidation, and ROS; upregulate transferrin and transferrin receptor; downregulate the SLC7A11 in a p53 dependent way	A549, SK, H661 and H460	Shao et al. (2022)
Solasonine	Suppress the expression of SLC711 and GPX4; affects mitochondrial function	Calu-1 and A549	Zeng et al. (2022)
		HepG2 and HepRG.	Jin et al. (2020)
		PANC-1 and CFPAC-1	Liang et al. (2022)
Ophiopogonin B	reduce the expression of GPX4 and SLC7A11	AGS and NCI-N87	Liyi Zhang et al. (2022)
Red ginseng polysaccharide	suppress the expression of GPX4	A549 and MDA-MB-231	Zhai et al. (2022)
Atractylodin	inhibit the expression of GPX4 and FTL proteins, and upregulate the expression of ACSL4 and TFR1 proteins	Huh7 and Hccm	Guan-Nan He et al. (2021)
Heteronemin	reduce the expression of GPX4	HA22T and HA59T	Chang et al. (2021)
Alloimperatorin	promote the accumulation of Fe^2+^, ROS and MDA, and reduce mRNA and protein expression levels of SLC7A11 and GPX4	MCF-10A, MDA-MB-231 and MCF-7	Liyi Zhang et al. (2022)
Tetrandrine citrate	suppress GPX4 expression and activate NCOA4-mediated ferritinophagy	MCF7 and MDA-MB-231	Yin et al. (2022)
Dihydroisotanshinone I	repress the protein expression of GPX4	MCF-7 and MDA-MB-231	Lin et al. (2019)
Saponin formosanin C	Inhibit SLC7A11 and GPX4	MDA-MB-231 and MCF-7	Hsin-Chih Chen et al. (2022)
*Lycium barbarum* polysaccharide	Inhibit SLC7A11 and GPX4	MDA-MB-231 and MCF-7	Du et al. (2022)
6-Thioguanine	inactivate System X_c_^−^, block the generation of GSH, downregulate the expression of GPX4, increase the level of lipid ROS	MGC-803 and AGS	Jinping Zhang et al. (2022)
Tanshinone IIA	upregulate p53 expression and downregulate SLC7A11 expression	BGC-823, NCI-H87 and BGC-823	Guan et al. (2020)
			Ni et al. (2022)
Jiyuan oridonin A derivative a2	Decrease GPX4 expression		Ying Liu et al. (2021)
Talaroconvolutin A	Increase ROS, downregulate SLC7A11 and upregulate arachidonate lipoxygenase 3	HCT116, SW480, and SW620	Xia et al. (2020)
Resibufogenin	Inactive GPX4	HT29 and SW480	Shen et al. (2021)
Drugs			
Orlistat	reduce the expression of GPX4 and induce lipid peroxidation	H1299 and A549	Wenjing Zhou et al. (2021)
Ketamine	Decrease the expression of GPX4	HepG2 and Huh7 MCF-7 and T47D	Guan-Nan He et al. (2021)
			Huixin Li et al. (2021)
Sulfasalazine	Increase ROS and decrease GPX4 and System X_c_^-^	MDA-MB-231 and T47D cells	Haochen Yu et al. (2019)
Metformin	reduce the protein stability of SLC7A11 by inhibiting its UFMylation process; downregulate GPX4 by targeting the miR-324-3p/GPX4 axis	MCF-7, T47D, HCC 1937, Bcap37, NHFB and HBL-100 MDA-MB-231	Jingjing Yang et al. (2021)
			Hou et al. (2021)
Actinidia chinensis Planch	inhibit the GPx4 and SLC7A11 proteins	HGC-27	Gao et al. (2020)
Cisplatin	Reduce GSH and inactive GPX4	A549 and HCT116	Guo et al. (2018)

Combination chemotherapy can significantly improve the efficacy and does not increase toxicity. SAS, an anti-inflammatory drug clinically used in ulcerative colitis, was found to activate ferroptosis in different breast cancer cells, especially in cells with low estrogen receptor expression (Yu H. et al., 2019). This results of the present study revealed that SAS could inhibit breast cancer cell viability, which was accompanied by an abnormal increase in ROS and a depletion of GPX4 and System X_c_^−^. Interestingly, in xenograft model, Polyphyllin III, a major saponin extracted from *Paris polyphylla* rhizomes, which induces Kruppel Like Factor 4-mediated protective upregulation of SLC7A11, in combination with the System X_c_^−^ inhibitor SAS, may have a co-induction in MDA-MB-231 breast cancer cells by enhancing intracellular lipid peroxidation and ferroptosis (Zhou Y. et al., 2021). Vorinostat, as mentioned before, is a histone deacetylase inhibitor (HDACI) has limited efficacy against solid tumors because of r the development of resistant cells (Fantin and Richon, 2007). SLC7A11 expressions positively correlate with insensitivity to HDACIs in many types of cancer cell lines (Fantin and Richon, 2007). Watanabe’ group demonstrated that the inhibition of SLC7A11 including SAS treatment may overcome resistance to vorinostat by accumulating ROS and inducing ferroptosis in human breast cancer cell and colon cancer cell (Miyamoto et al., 2020). Propofol is a short-acting intravenous anesthetic used for the induction and maintenance of general anesthesia. However, Propofol showed anti-proliferation effects on TNBC cells and could be a potential adjuvant to enhance the chemotherapeutic sensitivity of TNBC cells to doxorubicin and paclitaxel partly by promoting cell ferroptosis via p53-SLC7A11-GPX4 pathway (Sun et al., 2022). Additionally, siramesine, a lysosome disrupting agent, and lapatinib, an EGFR inhibitor, elicit ferroptosis in a synergistic manner in breast and lung cancer by altering iron regulation (Ma et al., 2016; Ma et al., 2017; Villalpando-Rodriguez et al., 2019).

### Liver cancer

To date, the main regulatory mediators mediating the ferroptotic response in Hepatocellular Carcinoma (HCC) cells have been identified as System X_c_^−^ and GPX4 (Zhang et al., 2020). The multikinase inhibitor Sorafenib is the conventional first-line chemotherapy drug used to treat advanced HCC. As a System X_c_^−^ inhibitor, Sorafenib is the only anticancer drug that causes ferroptosis in patients with HCC (Louandre et al., 2013). Although drug resistance limits its efficacy, it can still improve patient survival rates (Zhu Y.-J. et al., 2017). Interestingly, inhibition of ferroptosis can usually be detected once upon sorafenib resistance occurs. The resistance of hepatocellular carcinoma cells to chemotherapeutic agents such as sorafenib involves the abnormal expression of multiple transcription factors such as Nrf2, retinoblastoma (Rb) protein, hepatocyte nuclear factor 4α(HNF4α), HIC ZBTB Transcriptional Repressor 1 (HIC1), O-GlcNAcylated c-Jun, YAP/TAZ.

Nrf2 functions by blocking GSH depletion-mediated lipid peroxidation in HCC cells and plays a central role in protecting them from sorafenib-induced ferroptosis (Sun et al., 2016b). The status of Nrf2 is a key determinant of the effect of System X_c_^−^GSH/GPX4 axis-targeted therapy in HCC, and therefore it is necessary to improve efficacy by inhibiting Nrf2 expression. Nrf2 also counteracts sorafenib-induced ferroptosis by upregulating the iron and reactive oxygen metabolism-related genes HO-1 via the P62-Keap1-Nrf2 pathway (Sun et al., 2016b). Metallothionein-1G (MT-1G), a pivotal negative ferroptosis regulator, is a key regulator and promising therapeutic target for sorafenib resistance in human HCC cells. Sorafenib significantly induces exspression of MT-1G messenger RNA and proteins, and activation of Nrf2 is critical for MT-1G expression induced after sorafenib treatment (Houessinon et al., 2016). Importantly, the genetic and pharmacological inhibition of MT-1G enhances the anticancer activity of sorafenib *in vitro* and tumor xenograft models (Sun et al., 2016a). Sigma-1 receptor (S1R) is an oxidative stress-related protein, which regulates ROS accumulation via Nrf2. (Ha et al., 2011; Pal et al., 2012). Sorafenib significantly upregulated S1R protein expression in HCC cells. Studies have confirmed that the inhibition of S1R strengthen the anticancer effects of sorafenib in HCC cells *in vitro* and *in vivo* by inhibiting the expression of GPX4 (Bai et al., 2019). Overcoming the compensatory elevation of Nrf2 renders hepatocellular carcinoma cells more vulnerable to disulfiram/copper-induced ferroptosis (Ren et al., 2021). Meanwhile, the expression of Nrf2 is suppressed by Glutathione S-transferase zeta 1 (GSTZ1), which ia an enzyme in the catabolism of phenylalanine significantly downregulated in sorafenib-resistant hepatoma cells (Wang Q. et al., 2021a). Mechanistically, GSTZ1 depletion enhanced the activation of the Nrf2 pathway and increased the GPX4 level, thereby suppressing sorafenib-induced ferroptosis (Wang Q. et al., 2021a). But the combination of sorafenib and RSL3 significantly inhibited GSTZ1-deficient cell viability and promoted ferroptosis and increased ectopic iron and lipid peroxides *in vitro* and *in vivo* (Wang Q. et al., 2021a), The loss of function of the Rb protein (a tumor suppressor protein) is an important event during liver carcinogenesis, yet the mechanisms involved are complex. The high expression of RB protein in tumor cells inhibits ferroptosis by interfering mitochondrial ROS production, while also inhibiting the efficacy of sorafinib. Inhibiting RB protein *in vivo* promotes the efficacy of sorafenib, and RB protein can be used as an indicator of sorafenib sensitivity (Louandre et al., 2015). HNF4α has been identified as suppressing ferroptosis, and HIC1 identified as stimulating ferroptosis in liver cancer. HNF4A is critical for liver development (Parviz et al., 2003), and is up-regulated in liver cancer (Dill et al., 2013). By contrast, HIC1 acts as a tumor suppressor, which inhibits cell growth, migration and survival (Ubaid et al., 2018). Wang’group are the first to reveal that HNF4α and HIC1 oppositely regulate production of GSH via PSAT1, a key enzyme in GSH synthesis (Zhang et al., 2019). Increasing the concentration of GSH by targeting HNF4α and HIC1 might improve soferanib resistance for liver cancer treatment (Zhang et al., 2019). c-Jun is a regulator of glucose metabolism. Activation of c-Jun is associated with resistance to sorafenib and poor overall survival and inhibits sorafenib-induced cell death (Chen et al., 2016; Haga et al., 2017). Moreover, Chen et al. found that O-GlcNAcylated c-Jun stimulated GSH synthesis via increasing PSAT1 and CBS transcription to inhibit ferroptosis in HCC cells (Chen et al., 2019). YAP/TAZ are well-characterized transcriptional effectors of Hippo signaling involved in a variety of physio-pathological processes, including tumorigenesis and tissue regeneration (Harvey et al., 2013). Previous studies have suggested the Hippo-YAP/TAZ pathway is a key driver of ferroptosis in epithelial tumors (Harvey et al., 2013; Yang et al., 2019). However, Gao et al. revealed that YAP/TAZ as key drivers of Sorafenib resistance in HCC by repressing Sorafenib-induced ferroptosis (Gao et al., 2021). Mechanistically, YAP/TAZ induce the expression of SLC7A11 in a TEAD-dependent manner, and sustain the protein stability, nuclear localization, and transcriptional activity of ATF4 which in turn cooperates to induce SLC7A11 expression.

In addition to transcription factors mentioned above, there are also some other mechanisms involved in sorafenib resistance. Branched-chain amino acid aminotransferase 2 (BCAT2) is a novel suppressor of ferroptosis. Mechanistically, BCAT2 as the key enzyme mediating the metabolism of sulfur amino acid, regulated intracellular glutamate level, whose activation by ectopic expression specifically antagonize System X_c_^−^ inhibition and protected liver and pancreatic cancer cells from ferroptosis inducers (erastin, sorafenib, and SAS)-induced ferroptosis *in vitro* and *in vivo* (Wang K. et al., 2021). Besides, ATP-binding Cassette Subfamily C Member 5 (ABCC5), an important membrane transporter, is a universal glutamate conjugate that affects the efflux of endogenous metabolites, toxins, drugs, and intracellular ions (Jansen et al., 2015). The expression of ABCC5 was dramatically induced in sorafenib-resistant HCC cells and was remarkably associated with poor clinical prognoses (Huang et al., 2021). ABCC5 increased intracellular GSH and attenuated lipid peroxidation accumulation by stabilizing SLC7A11 protein, which inhibited ferroptosis (Huang et al., 2021). Additionally, the inhibition of ABCC5 enhanced the anti-cancer activity of sorafenib *in vitro* and *in vivo* (Huang et al., 2021).

DAZ Associated Protein 1 (DAZAP1) is an RNA-binding protein whose relative expression is significantly upregulated in HCC and is positively correlated with several key malignant features and poor postoperative survival in patients. Furthermore, DAZAP1 significantly reduced cellular sensitivity to sorafenib because DAZAP1 interacts with the 3′UTR (untranslated region) of SLC7A11 mRNA and positively regulated its stability (Wang Q. et al., 2021b). Protocadherin Beta 14 (PCDHB14), a member of the cadherin superfamily, is inactivated by aberrant methylation of its promoter in HCC patients and that PCDHB14 ablation inhibit cell cycle arrest, cell proliferation and ferroptosis (Liu Y. et al., 2022). Mechanistically, PCDHB14 is induced by p53, and increased PCDHB14 downregulates the expression of SLC7A11, which is mediated by accelerated p65 protein degradation resulting from PCDHB14 promoting E3 ubiquitin ligase RNF182-mediated ubiquitination of p65 to block p65 binding to the promoter of SLC7A11 (Liu Y. et al., 2022). Transforming growth factor β1 (TGF-β1) is a dichotomous cytokine that acts as a tumor suppressor in low-grade carcinoma cells but as a promotor of metastasis in advanced carcinoma cells (Dituri et al., 2019). Kim’ study was the first to show that TGF-β1 repressed the protein and mRNA levels of SLC7A11 in liver cancer cell lines with an early TGF-β1 gene signature but not in those with a late TGF-β1 gene signature (Kim et al., 2020). Macropinocytosis and transsulfuration pathway are important nutrient-scavenging pathway in certain cancer cells, allowing cells to compensate for intracellular amino acid deficiency under nutrient-poor conditions. Sorafenib increased macropinocytosis in human HCC specimens and xenografted HCC tissues, which prevented sorafenib-induced ferroptosis by replenishing intracellular cysteine that was depleted by sorafenib treatment; this rendered HCC cells resistant to sorafenib (Byun et al., 2022). Primary hepatocytes are able to survive for several days in the absence of Cys or cysteine in the culture medium, which thanks to the protective effect of transsulfuration pathway (Lee et al., 2017).

Some natural compouds that induce ferroptosis in liver cancer cells are summarized in Table 2. Some of them also have synergistic effects with sorafenib such as artesunate (Li Z.-J. et al., 2021), Dihydroartemisinin (Cui et al., 2022), Ursolic acid (Li H. et al., 2022), enhancing the anti-cancer effects of sorafenib. Moreover, The depletion of Phosphoseryl-tRNA kinase (PSTK) results in the inactivation of GPX4 and the disruption of GSH metabolism (Chen Y. et al., 2022a). Punicalin, an agent used to treat hepatitis B virus (HBV), was identified as a possible PSTK inhibitor that exhibited synergistic efficacy when applied together with Sorafenib to treat HCC *in vitro* and *in vivo* (Chen Y. et al., 2022a). Taken together, these results support the idea that blocking System X_c_^−^/GSH/GPX4 axis in combination with chemotherapeutic agents (e.g., sorafenib) provides new ideas for treatment of drug-resistant HCC.

### Gastrointestinal cancers

Gastric cancer is the fourth most common cancer worldwide and the second leading cause of cancer-related death after lung cancer (2020b). GPX4 is lowly expressed in gastric cancer (GC) cells, making them more susceptible to ferroptosis than normal intestinal cells. Cysteine dioxygenase 1 (CDO1) plays an important role in Erastin-induced ferroptosis in GC cells (Hao et al., 2017). CDO1 is a non–heme iron metalloenzyme, transforming cysteine to taurine by catalyzing the oxidation of cysteine to its sulfinic acid, which prevents cytotoxicity from elevated cysteine levels (Stipanuk et al., 2009). Suppression of CDO1 upregulates GPX4 expression, restores cellular GSH levels, prevents ROS generation (Hao et al., 2017). Zhao et al. reported that apatinib reduced the expression of GPX4, thereby inhibiting the proliferation of GC cells and multidrug-resistant GC (Zhao et al., 2021). Previous study showed that miR-375 can inhibit *Helicobacter* pylori-induced gastric carcinogenesis (Miao et al., 2014). MiR-375 reduced the stemness of GC cells *in vitro* and *in vivo* by directly targeting SLC7A11 (Miao et al., 2014). Elevated Growth/differentiation factor 15 (GDF15) level in serum and increased GDF15 expression in cancer tissues are reported in patients with various cancers, and associated with the poor prognosis of the patients (Welsh et al., 2001; Welsh et al., 2003). Recent research has showed that GDF15 regulate SLC7A11 expression, and GDF15 knockdown promote erastin-induced ferroptosis by repressing SLC7A11 expression and suppressing the function of System X_c_^−^ (Chen L. et al., 2020). The inhibition of Sirtuins 6 (SIRT6), a member of the Sirtuin family of NAD (+)-dependent enzymes, lead to the inactivation of the Keap1/Nrf2 signalling pathway and downregulation of GPX4, which overcomes sorafenib resistance by promoting ferroptosis in gastric cancer (Cai et al., 2021).

Another therapeutic target that regulates GC ferroptosis, SLC7A11, its inhibitor Erastin hampers the survival of GC (Sun et al., 2020). Some miRNAs, such as miR-489-3p and miR-375, can directly target SLC7A11 and trigger ferroptosis (Ni H. et al., 2021). Levobupivacaine, an amide-based local anesthetic, inhibits GC by upregulating miR-489-3p (Mao et al., 2021). A study showed that induction of ferroptosis by blocking Nrf2-Keap1 pathway also sensitizes cisplatin-resistant GC cells to cisplatin (Fu et al., 2021). The latest study suggests that Signal transducer and activator of Transcription 3 (STAT3)-mediated ferroptosis is associated with chemoresistance in gastric cancer (Ouyang et al., 2022). STAT3 a key oncogene with dual functions of signal transduction and transcriptional activation, which is hyperactivated in the formation of most human cancers and plays a critical role in cell proliferation, angiogenesis, metastasis, and immunosuppression (El-Tanani et al., 2022). Ouyang et al. demonstrates that STAT3 binds to consensus DNA response elements in the promoters of the GPX4, SLC7A11, and regulates their expression, thereby establishing a negative STAT3-ferroptosis regulatory axis in gastric cancer (Ouyang et al., 2022). However, additional important molecular mechanisms by which STAT3 regulates ferroptosis deserve further exploration.

Similar to GC cells, targeting the System X_c_^−^/GSH/GPX4 axis is an effective way to inhibit the growth of drug-resistant colorectal cancer (CRC). In colorectal cancer stem cells (CSCs), SLC7A11 is extremely expressed, with high GSH levels and low ROS levels, leading to their extreme vulnerability to ferroptosis (Zeuner et al., 2014). FAM98A (Family with Sequence Similarity 98 Member A), a microtubule-associated protein, plays a critical role in promoting resistance to 5-fluorouracil (5-FU) in CRC. The Enhanced expression of FAM98A recover 5-FU suppressed CRC cell proliferation both *in vitro* and *in vivo* by activating the translation of SLC7A11 in stress granules (He et al., 2022). However, In the xenograft model, the inhibition of GPX4 restrain tumor regrowth after discontinuation of 5-FU treatment (Zhang X. et al., 2022). Yang et al. found that high expression of KIF20A in CRC cells was associated with oxaliplatin resistance, and that resistance to oxaliplatin in CRC could be overcome by disrupting the KIF20A/NUAK1/PP1β/GPX4 pathway (Yang C. et al., 2021). In addition, Serine and arginine rich splicing factor 9 (SFRS9) can upregulate the expression of GPX4 by binding to GPX4 mRNA, which promote the growth of CRC, while SFRS9 knockdown significantly inhibited tumor growth in nude mice (Wang R. et al., 2021). Thus, GPX4 and/or SLC7A11 inhibition combined with chemotherapy or targeted therapy may be a promising therapy for CRC. *In vitro*, β-elemene (a ferroptosis inducer) in combination with cetuximab was shown to induce iron-dependent ROS accumulation, GSH depletion, lipid peroxidation, upregulation of HO-1 and transferrin, and downregulation of GPX4, SLC7A11 and other negative regulatory proteins in KRAS mutant CRC cells (Chen P. et al., 2020). *In vivo*, co-treatment with β-elemene and cetuximab inhibited KRAS mutant tumor growth and lymph nodes metastases (Chen P. et al., 2020).

### Pancreatic cancers

The main reason for the poor prognosis of PDAC is the late diagnosis of the disease and resistance to drugs that induce apoptosis (Chen X. et al., 2021b). Therefore, ferroptosis may provide an alternative strategy for killing PDAC cells and overcoming apoptosis resistance (Chen X. et al., 2021a; Yang G. et al., 2021). Gemcitabine (a nucleoside analogue of deoxycytoside) has been at the forefront of the past few decades as a cornerstone of PDAC treatment, despite its poor clinical efficacy.

Gemcitabine induces NF-κB activation and NOX-mediated ROS accumulation in PDAC cells. As a feedback mechanism, elevated ROS levels lead to Nrf2 activation and increased intracellular GSH, which resists treatment with gemcitabine (Manea et al., 2007; Lister et al., 2011). SLC7A11 disruption in PDAC cell lines strongly affects their amino acid and redox balance, and thus suppresses *in vitro* and delays *in vivo* their proliferative phenotype (Daher et al., 2019). Importantly, unlike disruption of other essential amino acid transporters, genetic ablation of SLC7A11 enhance susceptibility to cell death via ferroptosis (Daher et al., 2019). However, *in vivo* SLC7A11 knock out PDAC cells grew normally. Their further study showed that the presence of a cysteine/cystine shuttle between neighboring cells is the mechanism that provides redox and nutrient balance, and thus ferroptotic resistance in SLC7A11 knock out PDAC cells (Meira et al., 2021). Cysteine is required for preventing ferroptosis in pancreatic cancer (2020a), While the raw material for the synthesis of cysteine is mainly provided by System X_c_^−^.

Gemcitabine resistance was also associated with GPX4 in PDAC cells. Recent reports elucidated that HSPA5 upregulation negatively regulates ferroptosis in pancreatic cancer, while ATF4 activation upregulates HSPA5, thus the HSPA5-GPX4 pathway is one of the causes of gemcitabine resistance (Zhu S. et al., 2017). When gemcitabine was combined with HSPA5 inhibitor for PDAC, its anticancer activity was significantly enhanced (Zhu S. et al., 2017). Both rapamycin (classical autophagy inducer) and RSL3 can block mechanistic target of rapamycin kinase (MTOR) activation and cause GPX4 protein degradation in human pancreatic cancer cells, which can restore or enhance the anticancer activity of gemcitabine *in vitro* or in xenogeneic PDAC models (Liu Y. et al., 2021b). In addition, mitochondrial protease Lon peptidase 1 (LONP1) inhibits Nrf2-mediated GPX4 gene expression, thereby promoting Erastin-induced ferroptosis in human PDAC cells (Wang H. et al., 2020). However, the high-iron diets or depletion of GPX4 promotes Hydroxyguanosine 8 (8-OHG) release and thus activates the TMEM173/STING-dependent DNA sensor pathway, which results in macrophage infiltration and activation during Kras-driven PDAC in mice (Dai et al., 2020).

An emerging oncoprotein, Myoferlin, controls mitochondria structure and respiratory functions, has been associated with a low survival in several cancer types including PDAC. The pharmacological inhibitor of myoferlin can reduce the abundance of System X_c_^-^and GPX4 which trigger mitophagy and ROS accumulation culminating with lipid peroxidation and ferroptosis (Rademaker et al., 2022). The latest research shows that mitochondrial calcium uniporter (MCU) promotes PDAC cell migration, invasion, metastasis, and metabolic stress resistance by activating the Keap1-Nrf2 antioxidant program (Wang et al., 2022). SLC7A11 was identified as a druggable target downstream of the MCU-Nrf2 axis (Wang et al., 2022). But MCU overexpression makes PDAC cells hypersensitive to cystine deprivation-induced ferroptosis (Wang et al., 2022). Pharmacologic inhibitors of SLC7A11 effectively induce tumor regression and abrogate MCU-driven metastasis in PDAC (Wang et al., 2022). Natural compounds such as piperlongumine and cotylenin A exhibit synergistic therapeutic efficacy with SAS, which suggest that the triple combined treatment with piperlongumine, piperlongumine and SAS is highly effective against pancreatic cancer (Kasukabe et al., 2016; Yamaguchi et al., 2018).

In summary, System X_c_^−^/GSH/GPX4 axis-based ferroptosis may be a research direction for reversing drug resistance and may provide a rational basis for the development of new therapies for drug resistant solid tumors.

## Ferroptosis resistance in tumor cells

In addition to System X_c_^−^/GSH/GPX4 axis, an antioxidant system that protect tumor cells from ferroptosis, a number of new mechanisms of ferroptosis resistance have been found in *vivo* and *in vitro* studies of drug-resistant solid tumors mentioned above, which are associated with a variety of metabolic enzymes. Metabolic reprogramming is required for both malignant transformation and tumor development, including invasion and metastasis.

Lung adenocarcinomas select for expression of a pathway related to NFS1 that confers resistance to high oxygen tension and protects cells from undergoing ferroptosis in response to oxidative damage (Alvarez et al., 2017). NFS1 is an essential enzyme in eukaryotes that harvests sulfur from cysteine for the biosynthesis of iron–sulfur clusters, is particularly important for maintaining the iron-sulfur co-factors present in multiple cell-essential proteins upon exposure to oxygen compared to other forms of oxidative damage (Stehling et al., 2014). However, the specific mechanism is not yet clear. The latest *in vitro* and *in vivo* research results show that oxaliplatin-based oxidative stress enhance the phosphorylation level of serine residues of NFS1, which protect CRC cells in an S293 phosphorylation-dependent manner during oxaliplatin treatment (Lin et al., 2022). While NFS1 deficiency synergizing with oxaliplatin by increasing the intracellular levels of ROS (Lin et al., 2022).

Nuclear Protein 1 (NUPR1), a stress-inducible transcription factor, was identified as a driver of ferroptosis resistance through regulating Lipocalin 2 (LCN2) (Liu J. et al., 2021). LCN2, a secreted glycoprotein, forms a complex with bacterial and human siderophores, thereby inhibiting bacterial growth and regulating iron homeostasis that maintains the integrity of the gastrointestinal mucosa (Xiao et al., 2017). LCN2 expression is elevated in multiple tumor type. The overexpression of LCN 2 leads to resistance to 5-FU in CRC cell lines *in vitro* and *in vivo* by decreasing intracellular iron levels and stimulating the expression of GPX4 and SLC7A11 (Chaudhary et al., 2021).

Lymphoid-specific helicase (LSH) is involved in ferroptosis and is a potential therapeutic target in cancer because of its crucial role in ferroptosis. LSH, a member of the chromatin remodeling ATPase SNF2 family, establishes the correct levels and patterns of DNA methylation, maintains the stability of the genome in mammalian somatic cells, and is essential for normal development (Fan et al., 2003; Myant et al., 2011; Yu et al., 2014; Jia et al., 2017). One study showed that LSH lower the concentration of lipid ROS and iron by interacting with WDR76, activating lipid metabolic genes, including SCD1 and FADS2, which inhibit the accumulation of lipid ROS and intracellular iron (Jiang et al., 2017a). They further demonstrated that EGLN1 and c-Myc directly activated the expression of LSH by inhibiting HIF-1α (Jiang et al., 2017b). In addition, LSH promotes the expression of long non-coding RNAs LINC00336 in lung cancer tissues. LINC00336 acts as an oncogene that promotes tumor cell proliferation, inhibits ferroptosis, and induces tumor formation in an ELAVL1-dependent manner (Wang M. et al., 2019).

A cell-autonomous mechanisms have been identified that account for the resistance of cells to ferroptosis. The FSP1-CoQ10-NAD(P)H pathway exists as a stand-alone parallel system, which co-operates with System X_c_^−^/GSH/GPX4 axis to suppress phospholipid peroxidation and ferroptosis (Doll et al., 2019). Ferroptosis suppressor protein 1 (FSP1) previously called apoptosis-inducing factor mitochondria-associated 2 (AIFM2) confers protection against ferroptosis by complement the loss of GPX4. Furthermore, the suppression of ferroptosis by FSP1 is mediated by coenzyme Q10 (CoQ10), whose reduced form traps lipid peroxyl radicals that mediate lipid peroxidation, whereas FSP1 catalyses the regeneration of CoQ10 using NAD(P)H (Bersuker et al., 2019). Circular RNA circGFRA1 is remarkably upregulated in HER-2-positive breast cancer, which can bind to miR-1228 and alleviate inhibitory activity of miR-1228 on targeted gene AIFM2 (Bazhabayi et al., 2021). Knockdown of circGFRA1 could attenuate HER-2-positive breast cancer progression by inhibiting the proliferation, infiltration and migratory ability of HER-2-positive breast cancer cells. In addition, plasma-activated medium induces ferroptosis by depleting FSP1 in human lung cancer cells (Jo et al., 2022). Meanwhile, CoQ10-FSP1 axis is a key downstream effector of Keap1-Nrf2 pathway (Koppula et al., 2022). Keap1 is mutated in around 16% of NSCLCs (2012). Keap1 mutation or deficiency in lung cancer cells upregulates FSP1 expression through Nrf2, leading to ferroptosis- and radiation-resistance (Koppula et al., 2022). Furthermore, targeting the CoQ10-FSP1 axis sensitizes Keap1 mutant lung cancer cells or tumors to radiation by inducing ferroptosis (Koppula et al., 2022).

## Nanoparticles based on system X_c_^−^/GSH/GPX4 *axis* for treatment of drug-resistant solid tumors

The current ferroptosis inducers are mainly small molecules targeting the different targets in the System X_c_^−^/GSH/GPX4 axis. In mechanism, these inducers may have no cancer cell selectivity. Direct intravenous administration of these ferroptosis inducers of small molecules may lead new damage to normal cells and deteriorate the side effects of the current anti-tumor drugs. However, advances in nanomaterial sciences make it possible to improve the properties of drugs, prolong circulation times *in vivo*, and promote tumor-specific drug targeting to improve the therapeutic effect and reduce the incidence of adverse reactions (Amreddy et al., 2018). The nanoparticles developed in recent years that act on the five drug-resistant solid tumors discussed above are summarized in Table 3.

**TABLE 3:** Novel nano drug delivery systems inducing ferroptosis in solid tumors via System Xc-/GSH/GPX4 axis.

Nanoparticles	Loaded Drugs	Delivery Systems	Mechanism of Drug Release	Mechanism of Action	Cancer Cell Lines	Reference
mPEG-*b*-P (DPA-r-GC)	RSL3	intracellular-acidity-ionizable poly (ethylene glycol)-block-poly (2- (diisopropylamino) ethyl methacrylate) diblock copolymer and acid-liable phenylboronate ester dynamic covalent bonds	At neutral pH of 7.4, the nanoparticles can stably encapsulate RSL-3 inside the hydrophobic PDPA core via π–π stacking interaction with the phenylboronate ester groups; pH = 5.8–6.2, RSL3 release through acid-triggered cleavage of the phenylboronate ester dynamic covalent bonds and protonation of the hydrophobic core	Deplete system X_c_^−^	B16-F10 and 4T1	Song et al. (2021)
AMSNs/DOX	Doxorubicin	biocompatible arginine-rich manganese silicate nanobubbles	The positively charged drug binds to the negatively charged nanocarrier by electrostatic interaction, while the N atoms in the drug bind to the Mn atoms in the carrier by covalent bonding. At high GSH concentrations and low pH values, drug release is accelerated	Deplete GSH and inactive GPX4; release Mn ions and loaded drugs, resulting in enhanced T1-weighted magnetic resonance imaging contrast	Huh7 and L02	Wang et al. (2018)
MMSNs@SO	Sorafenib	manganese doped mesoporous silica nanoparticles	Manganese-oxidation bonds of nanocarrier could break in high GSH concentration, on-demand drug release is achieved due to the degradation of nanocarriers	consume intracellular GSH and inhibit system X_c_^−^	HepG2 and LO2	Tang et al. (2019)
FaPEG-MnMSN@SFB	sorafenib	manganese doped silica nanoparticle modified with folate grafted PEG	-Mn-O- bond in nanocarrier is sensitive to acidic and reductive environments and GSH can reduce the -Mn-O- bonds to Mn^2+^	consume intracellular GSH and inhibit system X_c_^−^	L02, HUVEC, HepG2, A549 and 4T1	Tang et al. (2020)
MIL-101(Fe)@sor	sorafenib	Fe-metal organic framework [MIL-101 (Fe)]	sorafenib gradually release in a time- and pH-dependent manner without an obvious burst-release effect. Drug release reached approximately 35% at pH 5.5 and only 10% at pH 7.4 after 60 h	consume GSH, decrease GPX4 levels, enhance lipid peroxidation generation, and simultaneously supply iron ions	HepG2	Xianchuang Liu et al. (2021)
AAAF@Cur	Curcumin	the hydrophilic end astragalus polysaccharides connect the ferrocene with azobenzene linker to construct the amphiphilic a hypoxia-responsive liver targeting carrier material AA/ASP-AZO-Fc (AAAF)	The azobenzene linker can be easily broken relying on the reduction reaction in a low oxygen environment, and then triggers the release of the drug	inhibit GSH content	HepG2	Xue Liu et al. (2022)
RSL3@O2-ICG NBs	RSL3	a 2-in-1 nanoplatform connected with nanobubbles (NBs) and sonosensitizer Indocyanine green	NBs could be used as cavitation nuclei, which may expand, compress and destroy under ultrasound stimulation. In cavitation, destruction generates microjets that create shear stress on cells and leads to reversible pore formation in the cell membranes, which could enhance cell membrane permeability transiently without deterring the cell viability and promote the drug into cells	consume GSH, inhibit GPX4 and cause ROS accumulation	HepG2 and Huh7	Yichi Chen et al. (2022b)
Erastin@FA-exo	Erastin	exosomes labeled with folate	Exosomes interact with cellular membranes and deliver drugs to cells	deplete GSH over generate ROS, suppress expression of GPX4 and upregulate expression of cysteine dioxygenase	MDA-MB-231	Mengyu Yu et al. (2019)
CSO-SS-Cy7-Hex/SPION/Srfn	sorafenib	Mitochondrial membrane anchored oxidation/reduction response and Fenton-Reaction-Accelerable magnetic nanophotosensitizer complex self-assemblies	The nano-device enrich the tumor sites by magnetic targeting of enhanced permeability and retention effects, which were disassembled by the redox response under high levels of ROS and GSH in ferroptosis therapy cells. Superparamagnetic iron oxide nanoparticles released Fe^2+^ and Fe^3+^ in the acidic environment of lysosomes, and the NIR photosensitizer Cy7-Hex anchored to the mitochondrial membrane, combined sorafenib leading to lipid peroxidation burst	deplete GSH over generate ROS, suppress system X_c_^−^ and enhance Fenton reaction	4T1, MCF-7, and MDA-MB-231	Sang et al. (2019b)
CSO-BHQ-IR780-Hex/MIONPs/Sor	sorafenib	Black Hole Quencher-cyanine conjugates based fluorescence “off−on” NIR nanophotosensitizer self-assembly chitosan with loaded magnetic iron oxide nanoparticles	Black Hole Quencher and IR780 are covalent binding via an ether bond, which is reduced by GSH. Subsequently, the IR780-Hex anchored the mitochondrial membrane nanoparticles and produce a large amount of ROS under a near-infrared laser. magnetic iron oxide nanoparticles release Fe^2+^ under an acid environment	suppress the SLC7A11, GPX4 system and lead to lipid peroxidation burst	4T1 and MCF-7	Sang et al. (2019a)
SRF@Hb-Ce6	sorafenib	a 2-in-1 nanoplatform connected with hemoglobin, the photosensitizer chlorin e6 and the amphiphilic matrix metalloproteinases 2-responsive peptide	Drug release is generally caused by matrix metalloproteinases 2-triggered cleavage, degradation, and/or dissociation of the nanomaterials	Reduce the expression of SLC7A11 and SLC3A2; downregulate GPX4	4T1, HepG2 and A549	Xu et al. (2020)
FPBC@SN	sorafenib and NLG919	The benzimidazole-cyclodextrin-switch-containing polymer vehicle	In weakly acidic solutions suchas tumor cells, protonated benzimidazole was transferred from hydrophobicity to hydrophilicity, and escaped from benzimidazole chambers to lead to nanoparticles disassembly	upregulate nuclear receptor coactivator 4, promote ferritinophagy, enhance Fenton reaction and immunotherapy, block glutathione synthesis and downregulate GPX4	4T1	Zuo et al. (2021)
HMTBF	Bleomycin and ML210	the metal-phenolic network formed by tannic acid, bleomycin, and Fe^3+^ with GPX4 inhibitor (ML210) loaded hollow mesoporous Prussian blue (HMPB) nanocubes	The nanoparticles degrade intracellularly to release drugs	Inactive GPX4, enhance Fenton reaction and apoptosis	4T1	Lulu Zhou et al. (2021)
Cu-TCPP(Fe) MOF	RSL3	Cu-tetra(4-carboxyphenyl) porphyrin chloride metal organic framework-based nanosystem modified with polyethylene glycol and iRGD	The nanosheet system release the supramolecular attached RSL3 in the acidic lysosomes	lead to the simultaneous inhibition of the GPX4/GSH and FSP1/CoQ10H2 pathways	4T1	Ke Li et al. (2022)
CDC@SRF	sorafenib	lipid-like dimersomes fabricated by cinnamaldehyde dimers	After reaching the tumor, the nanoparticles quickly underwent breakage in the cytosol owing to the conjugation of hydrophilic GSH on cinnamaldehyde dimers by Michael addition, which not only triggered the drug release	deplete intracellular GSH and inhibit system X_c_^−^	4T1	Zhou et al. (2022)

## Discussion and prospects

Ferroptosis is an iron-mediated form of cell death caused by the accumulation of lipid peroxides. The intracellular imbalance between oxidant and antioxidant due to the abnormal expression of multiple redox active enzymes will promote the produce ROS. Since the discovery of ferroptosis, much solid evidence has been obtained in experimental tumor models that ferroptosis has good anti-cancer effects. Many aggressive and drug-resistant cancer cells are sensitive to ferroptosis, so ferroptosis is indicated for those tumor cells that are less sensitive to chemotherapy. There is also growing evidence that the activation of ferroptosis contributes to the anticancer treatment of several forms of drug-resistant solid tumors, such as liver cancer, lung cancer, breast cancer, pancreatic cancer, gastrointestinal cancers, which undoubtedly makes that the combination of System X_c_^−^/GSH/GPX4 axis-based ferroptosis inducers with chemotherapeutic agents may become a new strategy for the treatment of drug-resistant solid tumors. The process of ferroptosis is a pharmacologically modifiable pathway, and there are many easy-to-treat targets on the System X_c_^−^/GSH/GPX4 axis.

The recognition of many natural products, and some drugs that have been marketed or used in the clinic were found to have the effect of inhibiting System X_c_^−^/GSH/GPX4 axis, thereby inducing ferroptosis. Many tumor cells develop chemoresistance through different mechanisms, including primary and acquired resistance. Acquired drug resistance is the most common cause of tumor recurrence. With the passage of time of drug treatment, spontaneous mutations from the tumor itself will lead to an increase in resistant clones. Because of the high heterogeneity of tumors, according to Darwinian evolutionary principles, drug-resistant mutants are selected, or subpopulations of primary resistant tumor stem cells in the dormant phase can cause tumors to regrow or spread. These drug resistant tumor cells protect themselves from death threats including ferroptosis inducers through many mechanisms including System X_c_^−^/GSH/GPX4 axis. GPX4 and GSH, as important intracellular antioxidants, protect cells from ferroptosis; therefore, GSH deprivation and GPX4 inactivation lead to ferroptosis. By inhibiting this system helps to promote tumor ferroptosis and alleviate drug resistance, which has also been confirmed in data from several studies. These drug-resistant solid tumors can be treated by the combination of ferroptosis inducers and other pathways. However, the induction of ferroptosis has a dual role in tumor growth. Some medicines induce ferroptosis to slow tumor growth, but ferroptosis itself can cause immunosuppression to accelerate tumorigenesis. So deeper mechanisms need to be explored. Also, considering that iron deficiency and iron overload may affect antitumor activity, the appropriate iron concentration and optimal dose of ferroptosis inducer medicines to reduce tumor progression deserves in-depth study. Furthermore, at present, all small molecules used in cancer targeting treatment finally produce drug resistance. The ferroptosis inducers of small molecules are no exception. Some cancer cells evolve specific mechanisms to resist ferroptosis, so discovering and inhibiting these mechanisms combined with ferroptosis is effective in alleviating drug resistance. Importantly, not all ferroptosis inducers are highly tumor-selective, so more research is needed to develop ways to improve drug targeting. Thorough studies are necessary to reveal effective multimodal therapies. Overall, inhibition of key molecules in System X_c_^−^/GSH/GPX4 axis is a promising therapeutic strategy for the treatment of tumors, especially drug-resistant tumors.

## Glossary

AREs: antioxidant response elements
AARE: amino acid response element
ATF3: Activating transcription factor 3
ATF4: Activating transcription factor 4
ABCC5: ATP-binding Cassette Subfamily C Member 5
AIFM2: apoptosis-inducing factor mitochondria-associated 2
BRD4: Bromodomain-containing protein 4
BSO: buthionine sulfoxide
BACH1: BTB and CNC homology 1
CGL: cystathionine γ-lyase
BCAT2: Branched-chain amino acid aminotransferase 2
CREB: cyclic adenosine monophosphate response element binding protein
CHaC1: Gamma-Glutamylcyclotransferase 1
CDO1: Cysteine dioxygenase 1
CRC: colorectal cancer
CSCs: colorectal cancer stem cells
CoQ10: coenzyme Q10
DUB: deubiquitinase
DAZAP1: DAZ Associated Protein 1
EGFR: Epidermal Growth Factor Receptor
EP300: E1A binding protein P300
GSH: glutathione
FSP1: Ferroptosis suppressor protein 1
GPX4: glutathione peroxidase 4
GCL: glutamate cysteine ligase
GSS: glutathione synthetase
GCLC: Glutamate-Cysteine ligase Catalytic Subunit
GCLM: Glutamate-Cysteine ligase Modifier Subunit
GC: gastric cancer
GSTZ1: Glutathione S-transferase zeta 1
GDF15: Growth/differentiation factor 15
H3K9: H3 lysine 9
HSPA5: Heat shock protein A family member 5
HDACI: histone deacetylase inhibitor
HCC: Hepatocellular Carcinoma
HNF4α: hepatocyte nuclear factor 4α
HIC1: HIC ZBTB Transcriptional Repressor 1
HBV: hepatitis B virus
IKE: imidazole ketone Erastin
IPP: isopentenyl pyrophosphate
Keap1: Kelch-like ECH-associated protein 1
KRAS: Kirsten Rat Sarcoma
LOX: lipoxygenase
LOOHs: lipid hydroperoxides
LCN2: Lipocalin 2: Lipocalin 2
LUAD: lung adenocarcinoma
LONP1: Lon peptidase 1
LCN2: Lipocalin 2: Lipocalin 2
LSH: Lymphoid-specific helicase
MDA: malondialdehyde
MUC1: mucin glycoprotein Mucin 1
m6A: N6 -methyladenosine
MT-1G: Metallothionein-1G
MTOR: mechanistic target of rapamycin kinase
MCU: mitochondrial calcium uniporter
Nrf2: nuclear factor erythroid 2-related factor 2
NUPR1: Nuclear Protein 1
OTUB1: OTU deubiquitinase, ubiquitin aldehyde-binding 1
PE: Piperazine Erastin
p53: Tumor protein p53
PLOOH: phospholipid hydroperoxide
PUFAs: polyunsaturated fatty acids
PdPT: palladium pyrithione complex
PCDHB14: Protocadherin Beta 14
PSTK: Phosphoseryl-tRNA kinase
RSL3: RAS-selective lethal small molecule 3
RCD: regulatory cell death
ROS: reactive oxygen species
RRM2: Ribonucleotide reductase regulatory subunit M2
Rb: retinoblastoma; System Xc-, cystine/glutamate antiporter
SLC7A11: Solute Carrier Family 7 Member 11
SLC3A2: Solute Carrier Family 3 Member 2
STYK1: serine threonine tyrosine kinase 1
SEPHS2: selenophosphate synthetase 2
S1R: Sigma-1 receptor
SIRT6: Sirtuins 6
STAT3: Signal transducer and activator of Transcription 3
SFRS9: Serine and arginine rich splicing factor 9
TrxR1: thioredoxin reductase 1
Trit1, tRNA isopentenyltransferase 1
TGF-β1: Transforming growth factor β1
USP7: Ubiquitin Specific peptidase 7
γ-GC: γ-glutamylcysteine
5-FU: 5-fluorouracil
8-OHG: Hydroxyguanosine 8
